# Unique Presentation of Bortezomib-Associated Thrombotic Microangiopathy Responsive to Therapeutic Plasma Exchange and Eculizumab Therapy

**DOI:** 10.3390/hematolrep14020018

**Published:** 2022-04-05

**Authors:** Robert C. Sterner, William Nicholas Rose

**Affiliations:** Department of Pathology and Laboratory Medicine, University of Wisconsin-Madison, Madison, WI 53792, USA; rsterner2@wisc.edu

**Keywords:** thrombotic microangiopath, TMA, Bortezomib, drug-induced TMA, therapeutic plasma exchange, eculizumab

## Abstract

Thrombotic microangiopathies (TMA) are a rare group of life-threatening hematological conditions characterized by thrombocytopenia and microangiopathic hemolytic anemia. Although our understanding of the pathophysiology and the availability of diagnostic testing has improved for primary TMAs, such as thrombotic thrombocytopenic purpura, the pathophysiology underlying secondary TMAs, including drug-induced TMAs (DITMAs), remains less clear. In this case report, we present the unique case of a patient with a history of multiple myeloma that presented four months after the initiation of bortezomib therapy with a bortezomib-associated TMA that responded to therapeutic plasma exchange (TPE) with plasma replacement and eculizumab therapy. This case demonstrates the possible utility of TPE with plasma replacement and eculizumab therapy in DITMA patients that fail to respond following a trial of holding the suspected medication.

## 1. Introduction

Thrombotic microangiopathies are a group of hematologic disorders that are associated with endothelial injury and activation of the coagulation cascade, resulting in microangiopathic hemolytic anemia (MAHA) and thrombocytopenia [1]. Although there are several precipitating causes of TMA, these disorders are characterized by widespread microvascular thrombosis resulting in end-organ injury [1,2]. Broadly, primary etiologies of TMA include autoantibodies targeting antigens on ADAMTS13 (thrombotic thrombocytopenic purpura), infections such as *E. coli* O157:H7, which produces Shiga toxin (hemolytic uremic syndrome), and mutations within the complement regulatory pathway (atypical HUS or complement-mediated TMA), while secondary causes include drug-induced TMA (DITMA), malignancies, and rheumatological disorders [1,2,3]. Clinically, patients may present with nonspecific symptoms, and thus, the initial evaluation should focus on confirming the MAHA and thrombocytopenia [1,2,3].

Importantly, several medications have been associated with drug-induced TMA (DITMA) [2,4]. The mechanisms of medications, including several chemotherapeutic agents triggering DITMA, are often unclear but may involve endothelial injury or, in some cases, antibody production [4,5]. Bortezomib is a proteasome inhibitor that is indicated in the treatment of patients with multiple myeloma or mantle cell lymphoma [2]. Three published case reports of bortezomib-induced TMA have been reported after treatment of bortezomib for 9–21 days in patients with multiple myeloma [4,6,7]. In this case report, we describe the unique presentation of bortezomib-induced TMA after treatment with bortezomib for approximately four months.

## 2. Case Presentation

A 73-year-old man with no significant past medical history presented to the emergency department with a three-and-a-half-month history of back pain with acute worsening. Computed tomography (CT) and magnetic resonance imaging (MRI) revealed innumerable osteolytic lesions throughout the spine, sacrum, and pelvis. Work-up was significant for elevated creatinine 1.89 mg/dL, calcium 11.1 mg/dL, hemoglobin 10.9 g/dL, lactate dehydrogenase (LDH) within normal limits, beta 2 microglobulin 10.0 mg/L, and a serum protein electrophoresis (SPEP) that demonstrated a monoclonal IgG kappa and a serum kappa of 24.8 mg/dL for a kappa-to-lambda ratio 24. Subsequent biopsy confirmed a plasma cell neoplasm consistent with multiple myeloma revised international staging system (R-ISS) stage III.

**Figure 1:**
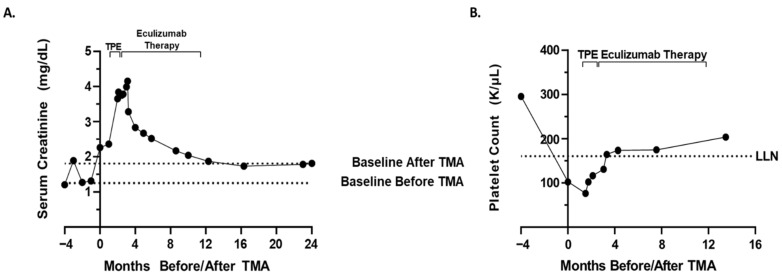
Serum creatinine (**A**) and platelet count (**B**) prior to and post eculizumab therapy and therapeutic plasma exchange. (**A**) Serum creatinine values are depicted prior to and following therapeutic plasma exchange (TPE) and eculizumab therapy. Therapy durations of TPE and eculizumab therapy are demarcated by black brackets. The dotted lines represent the patient’s baseline serum creatinine before and after thrombotic microangiopathy (TMA). (**B**) The patient’s platelet counts are shown prior to and after TPE and eculizumab therapy. Therapy durations of TPE and eculizumab therapy are demarcated by brackets. The dotted line represents the reference range lower limit of normal (LLN). Month zero corresponds to the date of onset of TMA, while months with a negative sign denote months prior to TMA.

The patient was treated with four cycles of bortezomib (2.3 mg/dose; 28-day cycle; one dose on day 1,8,15), lenalidomide (25 mg/dose; 28-day cycle; one dose on day 1,21), and dexamethasone (40 mg/dose; 28-day cycle; one dose on day 1,8,15,22) (VRd). After the 13th dose of bortezomib/fourth cycle of VRd therapy, the patient’s creatinine rose to 2.26 mg/dL from a baseline of 1.2 mg/dL prior to therapy (Figure 1A). The rise in creatinine directly correlated with the administration of VRd therapy, prompting a pause in VRd therapy and further work-up. Urinalysis showed proteinuria with 24 h urine protein elevated to 2.31 g from a baseline of 0.8 g prior to therapy and an elevated protein–creatinine ratio of 4.94 mg/mg from 2.31 mg/mg prior to the 13th dose of bortezomib. Urinalysis also showed hematuria, with 3+ urine hemoglobin and 20–51 red blood cells with normal morphology per high-powered field.

**Figure 2:**
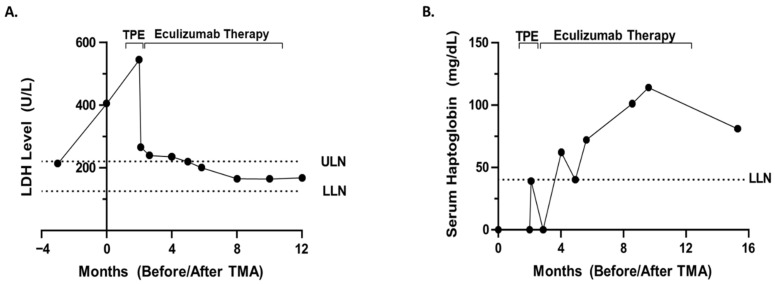
Lactate dehydrogenase and serum haptoglobin levels prior to and post eculizumab therapy and therapeutic plasma exchange. (**A**) Lactate dehydrogenase values are depicted prior to and following therapeutic plasma exchange (TPE) and eculizumab therapy. Therapy durations of TPE and eculizumab therapy are demarcated by black brackets. The dotted lines represent the LDH reference range upper and lower limits of normal (ULN, LLN). (**B**) The patient’s serum haptoglobin levels are shown prior to and after TPE and eculizumab therapy. Therapy durations of TPE and eculizumab therapy are demarcated by brackets. The dotted line represents the reference range lower limit of normal (LLN). Month zero corresponds to the date of onset of TMA, while months with a negative sign denote months prior to TMA.

Additional work-up was significant for complete blood count (CBC) showing a decrease in hemoglobin, 7.3 g/dL from 9.5 g/dL, prior to the 13th dose of bortezomib, platelet count 102 K/μL from 295 K/μL at baseline prior to therapy (Figure 1B), elevated lactate dehydrogenase of 405 U/L (Figure 2A), undetectable or low <8 mg/dL serum haptoglobin (Figure 2B), and ADAMTS13 activity level 47%. Peripheral blood smear revealed the presence of schistocytes and helmet cells consistent with a microangiopathic hemolytic process. No images of the peripheral smear are available. Kidney ultrasound revealed no evidence of hydronephrosis or concerning findings. Renal biopsy was ordered to confirm TMA versus acute interstitial nephritis versus renal insufficiency secondary to multiple myeloma.

Due to the global COVID-19 pandemic, renal biopsy was delayed. Bortezomib therapy was held, and the patient completed one cycle of lenalidomide and dexamethasone therapy. The patient’s renal function continued to worsen over the next month and a half, with an elevated creatinine of 3.65 mg/dL, up from 2.26 mg/dL. Given the patient’s worsening renal function, he was admitted for renal biopsy and TPE. Renal biopsy ultimately confirmed TMA most likely secondary to bortezomib. The patient underwent five procedures of one volume centrifugal plasmapheresis with plasma as the replacement fluid over the first seven days. Following TPE, there was improvement in the patient’s markers for hemolysis, including haptoglobin 39 mg/dL from <8 mg/dL and LDH 266 U/L from 545 U/L. TMA functional panel (Table 1) from the University of Iowa revealed low factor H level 134 mg/L (reference range: 180–420 mg/L) and mildly elevated Bb fragment level of 2.9 mg/L (reference range <2.2 mg/L). Molecular genetic testing for common mutations in complement TMA was ordered but was not covered by insurance, causing the patient to decline testing.

Renal function did not significantly improve but remained relatively stable following TPE. After the initial five procedures of TPE, the patient was weaned to TPE twice weekly and then once weekly. Due to the fact that the patient was dependent on TPE for hemolysis control and the mildly reduced factor H level, eculizumab therapy (1200 mg every two weeks) was initiated prior to stopping TPE. Overall, the patient underwent 16 TPEs with plasma replacement over the course of approximately two months. Eculizumab therapy resulted in improvement in renal function to a stable baseline creatinine of approximately 1.7 mg/dL and improved markers of hemolysis with a haptoglobin 81 mg/dL and LDH 168 U/L. Eculizumab therapy was stopped after nine months after the patient’s renal function/markers of hemolysis returned to baseline/were within normal limits. Overall, he received 1200 mg every two weeks for about nine months. Due to the concerns of renal insufficiency, the patient’s chemotherapy was switched to daratumumab 1800 mg. Currently, the patient appears to be in a complete remission with daratumumab.

## 3. Discussion

This case report presents a rare case of bortezomib-associated TMA in which bortezomib administration likely unmasked a hereditary atypical HUS (aHUS) that uniquely presented four months after initiating bortezomib therapy and was responsive to TPE with plasma replacement and eculizumab therapy. Bortezomib prevents NFκB activation through selective inhibition of the 26S proteasome and is approved in the treatment of multiple myeloma and mantle cell lymphoma [2,4,8]. Although thrombocytopenia was reported to occur in 30% of patients treated with bortezomib, these reports do not mention renal dysfunction as an adverse side effect and classify the thrombocytopenia as typically mild [2,9]. Three other cases of probable bortezomib-induced TMA have been reported in multiple myeloma patients [4,6,7]. Two additional cases of TMA in multiple myeloma patients taking bortezomib were complicated by and potentially confounded by a history of allogeneic hematopoietic stem-cell transplant and subsequent use of calcineurin inhibitors [2,10,11].

In the case presented above, in addition to bortezomib-associated TMA, the differential diagnosis for this patient’s worsening renal function included atypical HUS, acute interstitial nephritis, multiple myeloma itself as the underlying cause of TMA, renal dysfunction due to multiple myeloma itself secondary to variable cast nephropathy or tubular injury due to immunoglobulin light chains, TTP, acute tubular necrosis, autoimmune HUS, and prerenal etiologies. Due to the anemia, thrombocytopenia, peripheral blood smear findings consistent with MAHA, biopsy confirming TMA, ultrasound findings, elevated LDH, undetectable/low haptoglobin, and ADAMTS13 activity level 47%, the most likely diagnosis was bortezomib-associated TMA with bortezomib unmasking a hereditary aHUS. This diagnosis is also supported by the fact that the administration of bortezomib directly correlated with rising creatinine (Figure 1A), decreased the platelet count (Figure 1B), increased the markers of hemolysis (Figure 2), a peripheral blood smear showing microangiopathic hemolytic anemia, and other signs/symptoms of TMA. The diagnosis is further supported by the fact that a two-month trial of holding bortezomib did not restore renal function/improve markers of hemolysis. Finally, the patient’s renal function and markers of hemolysis only improved to baseline (Figure 1 and Figure 2) after treatment with eculizumab. Thus, the improvement with complement blockade further supports the most likely diagnosis of a bortezomib-associated TMA in which bortezomib unmasked a hereditary aHUS. On the other hand, the patient’s normal soluble C5b-9 level is not completely supportive of this hypothesis, and although molecular genetic testing was not possible for the patient in this case, molecular genetic testing is necessary in order to definitively conclude whether a patient has a hereditary aHUS. Therefore, although a bortezomib-associated TMA in which bortezomib unmasked a hereditary aHUS is the most likely diagnosis, this diagnosis cannot be concluded with complete certainty.

This case of bortezomib-associated TMA is unique, in that it is the first reported case of bortezomib-induced TMA to present at more than 21 days after initiating bortezomib therapy (four months after initiating bortezomib). The suspected drug, bortezomib, was discontinued. Approximately two months after discontinuing bortezomib, the patient’s renal function and hemolysis labs continued to worsen and prompted TPE with plasma exchange and eventually eculizumab. Two cases have reported improving renal function and improved hemolysis markers after discontinuing bortezomib and plasma exchange with one [4] and fourteen [7] daily TPE sessions, respectively. As is often the case in DITMA, the efficacy of TPE in patients from these two cases is unclear, as there was not a significant period of time between discontinuation of bortezomib and TPE initiation. After one month of holding bortezomib in the case presented above, TPE with plasma replacement improved hemolysis markers but did not improve renal function. The transition to eculizumab therapy for nine months resulted in improvement in renal function and hemolysis markers.

The key principle in managing patients with TMA is to identify the precipitating factor of the TMA syndrome [1,2,3]. For instance, in the case of patients with TTP, ADAMTS13 is deficient. Thus, plasma exchange is indicated to remove the offending autoantibody to ADAMTS13 and replenish the enzyme. In the case of DITMA, management has focused on discontinuing the triggering medication and supportive care [1,2,3].

Patients with TMA, however, may present with non-specific symptoms and have a variety of presentations, such as unexplained thrombocytopenia or anemia, unexplained neurological symptoms, or an acute illness [3,12]. Therefore, it is critical to know when potentially lifesaving plasma exchange is indicated if the clinician is awaiting ADAMTS13 test results, or if the etiology is not clear, but TTP is suspected. Recently, a systematic review involving nearly 1000 patients validated the diagnostic accuracy of the PLASMIC score, which predicts ADAMTS13 deficiency in patients with suspected TTP [13]. More specifically, the PLASMIC score assigns one point for each of the following seven features: (1) platelet count < 30 × 10^9^/L, (2) hemolysis, (3) absence of active cancer, (4) absence of history of solid-organ or stem-cell transplant, (5) mean corpuscular volume < 90 fL, (6) INR < 1.5, and (7) creatinine < 2.0 mg/dL [14]. The score ranges from 0–7, and a score of 5 or higher has a 99% sensitivity and 57% specificity for deficiency of ADAMTS13 [14]. Thus, these patients are recommended to undergo urgent TPE while awaiting ADAMTS13 test results. It is important to note that many hospitals, especially academic centers, now have the ability to rule out TTP with in-house ADAMTS13 testing within 45 min to a few hours due to the increased availability and distribution of test kits. Therefore, if a patient has a low PLASMIC score or a pretest probability for ADAMTS13 deficiency, then TTP can now be ruled out more rapidly with in-hospital testing and if clinically indicated, plasma can be administered while awaiting the results.

Although TPE has a clear, potentially lifesaving role in patients with TTP, the role of TPE in patients with secondary TMA due to DITMA is controversial. More specifically, the outcomes of DITMA have been variable, with some sporadic reports supporting the efficacy of TPE [15,16]. For instance, in patients with cyclophosphamide-induced microangiopathic hemolytic anemia, TPE has been associated with improved renal function [4,17]. Additionally, in patients with clopidogrel-induced TTP, TPE has been associated with decreased mortality [18]. The variable outcomes of TPE in DITMA likely stems from the underlying pathophysiology in which two distinct mechanisms: (a) an immune-mediated mechanism and (b) a dose-dependent toxicity mechanism have been identified [2].

Previous reports and studies have suggested that both dose-dependent and immune-mediated mechanisms are likely responsible for protease inhibitor (PI)-induced TMA. One postulated mechanism is that PIs such as bortezomib damage the endothelium by causing direct microvascular toxicity [2]. Other studies have suggested the PIs cause microvascular injury to the glomerular capillaries through their action via NFκB on the vascular endothelial growth factor (VEGF) pathway [19]. An immune-mediated mechanism of action for PI-associated TMA has also been proposed in which PI use results in high levels of proinflammatory cytokines, such as IL-6 and TNF- α [11]. These high levels of proinflammatory cytokines may then facilitate the production of autoantibodies directed against ADAMTS13 [2,11]. Although an immune-mediated mechanism could possibly be responsible for the decreased level of ADAMTS13 (47%; reference range: ≥60%) as seen in the case described above, given that the ADAMTS13 level is still well the 10% that is usually seen in TTP, PI-induced production of antibodies against ADAMTS13 is an unlikely explanation for the clinical picture presented. Theoretically, the production of antibodies that react with platelet–glycoprotein complexes as seen in patients with quinine-associated TMA could be possible; however, to date, there are no assays available to detect PI-dependent antibodies or reports of PIs inducing TMA via this mechanism [2,20].

Yui et al. [2] classified the previous three reports of probable bortezomib-induced TMA as immune mediated based on the patients’ rapid development of symptoms within 21 days after bortezomib was initiated. In this report, we presented a case of probable bortezomib-induced TMA presenting approximately four months after the patient’s first dose of bortezomib. On the one hand, if one considers only the timing of TMA onset, then an immune-mediated etiology is unlikely. On the other hand, due to the fact that this patient’s markers of hemolysis improved with (a) TPE with plasma replacement and (b) treatment with eculizumab, which prevents the activation of the terminal complement system through inhibition of cleavage of C5 into C5a/c5b, there appears to be at least a component of immune-mediated TMA in this patient.

## 4. Conclusions

This case report is, to our knowledge, the first to report a case of bortezomib-associated TMA after approximately four months of bortezomib therapy that responded to TPE with plasma replacement and eculizumab therapy. Although to date, all reports of bortezomib-associated TMA have presented within 21 days of initiation of bortezomib therapy, there are multiple reports of delayed onset of DITMA several months after initiating another proteasome inhibitor, carfilzomib [2]. While the role of TPE remains controversial, some immune-mediated mechanisms of PI-associated TMA could theoretically benefit from TPE with plasma replacement and/or eculizumab, as seen in this case. Importantly, although a subset of patients may benefit from TPE and/or eculizumab, the fundamental principle in managing DITMA is discontinuation of the triggering agent and supportive care. As presented above, in the population of patients with DITMA with renal dysfunction and hemolysis that fail to improve after discontinuation of the suspected medication, TPE with plasma replacement and eculizumab therapy may be considered.

## Figures and Tables

**Table 1:** TMA functional panel results. Complement biomarker profiling revealed a low FH level, an elevated Bb factor level, and the C3 level, C4 level, FB level, soluble C5b-9, and FI level were all within normal limits.

Test	Reference Range	Result	Interpretation
CH50eq	199 U Eq/mL	>70 U Eq/mL	Normal
Alternative Pathway Functional Assay	74%	50–130%	Normal
Hemolytic Assay	1.7%	<3%	Normal
Factor H Autoantibody	<50 AU	Titer < 200 AU	Negative
C3 Level	1.0 g/L	0.9–1.8 g/L	Normal
C4 Level	0.34 g/L	0.15–0.57 g/L	Normal
Factor B (FB) Level	29.1 mg/dL	22–50 mg/dL	Normal
Bb Fragment Level	2.9 mg/L	<2.2 mg/L	Elevated
Soluble C5b-9 (sMAC)	0.27 mg/L	<0.3 mg/L	normal
Factor H (FH) Level	134 mg/L	180–420 mg/L	Low
Factor I (FI) Level	35.7 mg/L	18–44 mg/L	Normal

## Data Availability

Not applicable.
